# Prediction of promiscuous epitopes in ORF2 of Hepatitis E virus: an *In-Silico* approach

**DOI:** 10.4314/ahs.v22i3.67

**Published:** 2022-09

**Authors:** Noor Samavia, Parvaiz Fahed, Waheed Yasir, Anwar Tasneem, Nasreen Syeda

**Affiliations:** 1 Department of Biosciences, COMSATS University, Islamabad, Pakistan; 2 Foundation University Medical College, Foundation University Islamabad, Pakistan; 3 IBADAT International University, Islamabad, Pakistan

**Keywords:** Virology, gastrointestinal disease

## Abstract

**Background:**

Vaccine development against emerging infections is essentially important for saving people from increasing viral infections. In developing countries, Hepatitis E (HEV) is a common infection affecting millions of people worldwide. Based on In-silico analysis, different approaches have been targeted.

**Objectives:**

Rationale of this study is to design an epitope-based vaccine candidates with the help of immunoinformatics that can predict promiscuous B-cell and T-cell epitopes of the most antigenic HEV-ORF2 capsid protein.

**Materials & Methods:**

This study suggests potential T-cell and B-cell epitopes of the highly antigenic HEV ORF2 capsid protein while using various In-silico tools such as NCBI-BLAST, Expassy, CLC workbench, Ellipro and Discotope.

**Results:**

Potential antigenic and immunogenic CD8+ T-cell epitopes were predicted from the global consensus sequence of ORF2-HEV. Furthermore, twenty-two linear B-cell epitopes were predicted. Among these, “SLGAGPV” at position 587–593 and “LEFRNLTPGNTNTRVSRYSS” at position 306–325 were most antigenic with antigenicity score 1.4206 and 1.3600 respectively. Discontinuous B-cell epitopes were found by three-dimensional capsid protein structure. Epitopes predicted in this study reveal high antigenicity and promiscuity for HLA classes.

**Conclusion:**

Collectively, our data suggests promiscuous epitopes that can potentially acts as new candidates for the design of HEV peptide vaccine.

## Introduction

Hepatitis E Virus (HEV) is a leading gastro-enteric viral infection that occurs worldwide. HEV shows strong hepatotropism and triggers acute infection, if gets untreated 1. HEV follows oral-faecal contamination and concentrates in areas of poor hygiene. As a result, there are frequently sporadic cases and periodic huge outbreaks occur. HEV-3 and HEV-4 are zoonotically transmitted from animal water sources in advanced countries, with periodic cases that have progressively more been revealed 2.

HEV poses silent yet major source of viral hepatitis in the world. HEV infection is worldwide in distribution. In developing countries, HEV is not generally considered as a serious disease and public awareness is lacking about this disease. According to a WHO survey, about 20 million cases of HEV are reported worldwide annually. Around, 60,000 individuals per year suffer from acute infections of HEV. However, the estimated mortality rate is even higher for pregnant women. During the summer, outbreak is more likely to occur. HEV mostly infects men ranging from 15–30 years of age and quite uncommon in children below the age of 10 3–4. The infection tends to have adverse effects for pregnant women, and is linked to greater risk of attack, elevated severity of the disease and a 15–25% morbidity ratio 4. Generally, HEV infection has been recognized in advanced countries by travellers returning from countries where it is common. It also reported the sporadic indigenous cases of HEV genotypes 3 and 4 in the past decade to endemic country without recent travel history and is more likely to preferred in middle-aged elderly men over the age of 40 5.

HEV is a positive ssRNA virus with a diameter of 27–34nm. The genome is organized into three ORFs (ORF1, ORF2, and ORF3) and short non-coding regions at both 5’ and 3’ ends 6–7. The ORF1 is involved in viral replication and protein processing by RNA-structured RNA polymerase. ORF2 contains capsid protein that is bound to the host cell and is responsible for selection of neutralizing antibodies. ORF3 involves in morphogenesis and virion release 8.

Nowadays, epitope-based strategies are one of the most effective strategies towards the development of vaccines. This approach was first introduced by Jacob in 1985 to develop a cholera vaccine 9. The basic idea beyond the development of epitope-based vaccines is to find immuno-dominant epitopes that are expected to elicit immune responses against a target pathogen in a given protein sequence 10. Humoral immune responses to HEV that demonstrated that pORF2 and pORF3 contain an excellent antibody mediated response directed against immunodominant antigenic epitopes have been studied 11–16. This study targets ORF2-HEV for the prediction of promiscuous epitopes.

Since HEV has the potential to get transformed into acute infections that can lead to hospital-based treatment. Therefore, there should be a proper vaccine available for this viral infection. However, HEV vaccine has been licensed in China only but not available elsewhere. Therefore, need of the hour is to design effective vaccine candidates against HEV through immunoinformatics based tools that should be validated with the wet lab analysis to make it available for everyone.

## Materials and Methods

### Retrieval of the target sequence

Homologous sequences of HEV-ORF2 were obtained from National Center for Biotechnology Information (NCBI). The nucleotide sequences were subjected to translation using EXPASY tool. (Reference Protein ID: HEVgp10 capsid protein, gene ID: 1494410).

### Development of global consensus sequence and Phylogenetic tree

Eighty-five protein sequences were subjected to multiple sequence alignment using CLC workbench (version 8.1) and a global consensus sequence, which is used for epitope prediction, was obtained. Furthermore, a phylogenetic tree was constructed for all the translated sequences using the CLC workbench.

### Epitope Prediction

Using the global consensus sequence of HEV ORF2, promiscuous T-cell and B-cell epitopes were predicted through various online bioinformatics tools.

### T-Cell Epitope prediction

Peptides binding to MHC-I and MHC-II molecules were predicted using the NetCTL1.2 server and IEDB tool respectively. For 12 supertypes such as A1, A2, A3, A24, A26, B7, B8, B27, B39, B44, B58 and B62, the NetCTL 1.2 server predicted CD8+ T-cell epitopes to be considered for MHC II binding. Peptides binding to MHC-II were predicted for HLA DRB1* allele(s): HLA DRB1*0101 and HLA DRB1*0701. The epitopes were analysed by the MHCPred 2.0 server to verify the binding affinity.

### Prediction of Linear B-cell epitopes

Linear B-cell epitopes were predicted via the online BepiPred-2.0 and BCpred linear epitope prediction method using IEDB. VaxiJen 2.0 was used to check the antigenicity of these linear B-cell epitopes.

### Prediction of conformational B-cell epitopes

To predict the B-cell epitopes, Ellipro and DiscoTope tools were used. ElliPro is the most reliable tool since it can model both linear and discontinuous epitopes based on the three-dimensional structures of protein. In addition, Discotope tool was used to analyze the surface's availability and score for epitope propensity. The higher score corresponds to the greater chance of the residue being involved in an epitope.

### Three-dimensional analysis of Capsid protein

In this study, HEV capsid protein crystal structure was visualized with PDB-id 3iyo. This database contains the compounds derived from the X-ray crystallography and NMR experiments. However, chimera was used to perform structural analysis of the protein.

## Results

### Multiple sequence alignment

**Figure 1:**
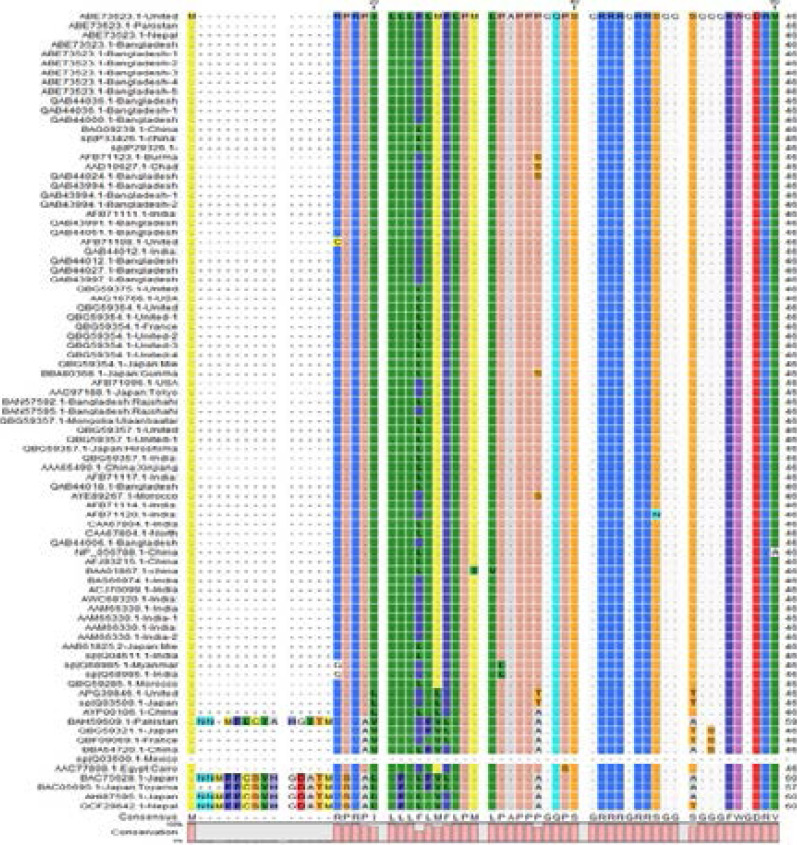
Multiple sequence alignment.

Sequence alignment of capsid protein sequence revealed 660 amino acids with highly conserved protein sequence while certain regions showed variations. Conserved regions shown with dots (Figure 1). ORF2 capsid protein is made up of three domains, the S domain (129–319), the M domain (320–455), and the P domain (456–606). In ORF2, at position 137, 310, 562 are the putative glycosylation sites; however, there were some mutations such as addition of amino acids or substitution of amino acids that could also be seen in sequence alignment. At position137 which is the putative glycosylation site, Proline (P) is replaced by Serine (S). At position 16, Arginine (A) is replaced by Cysteine(C) and Glycine(G). At position 24, Phenylalanine (F) is replaced by Leucine (L). At position 31, Leucine was replaced by Valine (V). At position 36, Proline (P) was replaced by Serine (S) and Threonine (T). At position 48, Serine (S) was replaced by Asparagine (N). At position 53, Glycine (G) was replaced by Serine (S). Substitution of amino acids could also be seen in capsid protein domains. There were also some empty positions where no amino acid is present and at these positions, amino acids were added leading to mutation. But these mutations do not significantly affect the viral replication or synthesis of capsid protein.

### Phylogenetic Tree

**Figure 2:**
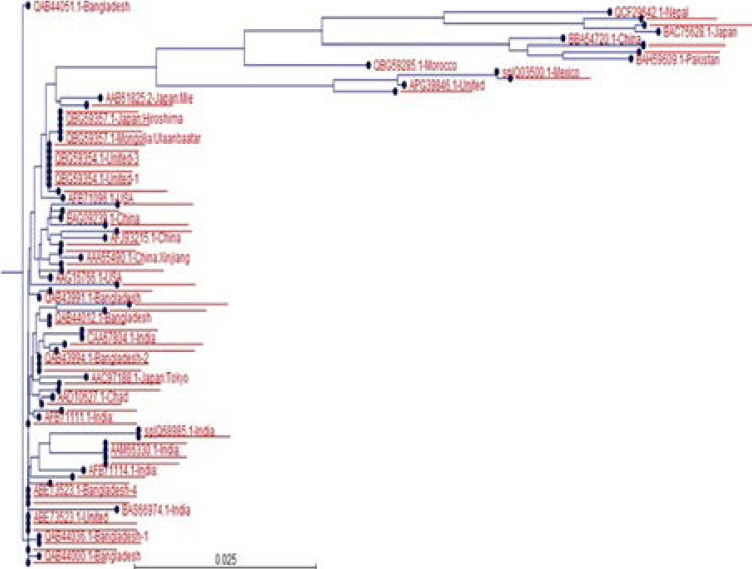
Phylogenetic tree.

A phylogenetic tree was constructed from eighty-five protein sequences (ORF2-HEV) across the world using CLC workbench (Figure 2). In this tree, the first branch makes a cluster containing Nepal, Japan, China, Pakistan, Morocco, Mexico and United States. Although there is a lot of sequence similarity because of conserved capsid protein sequence but sequence variation can also be seen among different strains or regions.

### CD8+ T cell Epitopes

CD8+ T-cell epitopes were predicted using NetCTL 1.2 tool. In this study we predicted total 20 epitopes which met the threshold value of 0.75 and demonstrated maximum interaction with all supertypes. The predicted T-cell epitopes were further evaluated for antigenicity and immunogenicity by the Vaxijen tool and the IEDB immunogenicity predictor. Here, 10 epitopes were below the threshold value of 0.4 on the Vaxijen server, out of 20 primarily selected epitopes, while 5 epitopes achieved an immunogenicity value of less than 0.00. Thus among 20 epitopes, 5 epitopes with positive antigenicity and immunogenicity scores have been selected (Table 1). These highly antigenic epitopes can bind with MHC alleles with high affinity and can provide an efficient immune response. A fair criterion for selecting a suitable epitope could be the immunogenicity score of the epitope.

**Table 1:** CD8+ T-cell Epitopes

	EPITOPES	HLA SUPERTYPES/ALLELES	ANTIGENECITY SCORE	IMMUNOGENECITY SCORE
**1.**	WLSLTAAEY	A1, B62	1.5019	0.10129
**2.**	IMATEASNY	A1, A3, B58, B62	0.7715	0.4269
**3.**	DSQPFAIPY	A1, A26, B62	0.7441	0.20971
**4.**	ESRVVIQDY	A1, A26	0.6128	0.13902
**5.**	LTTTAATRF	A1, B58	0.5458	0.18959

### CD4+ T-cell epitopes

Antigenic Epitopes “WLSTAAEY” “IMATEASNY” “DSQPFAIPY” “ESRVVIQDY” and ‘LTTTAATRF” were selected for the identification of MHC-II alleles and their corresponding peptide or CD4+ T-cell epitope. The analysis revealed that peptides WLSTAAEYDQSTYG at position 472–486, IMATEASNYAQYRVV at position 174–188, DSQPFAIPYIHPTNP at position 47–61 and ESRVVIDQYDNQHEQ at position 435–449 showed affinity with HLA DRB1* allele(s): HLA DRB1*0101 and HLA DRB1*0701. Therefore, the strongest binding grooves for the predicted peptides may be HLA DRB1* alleles. For the effective functioning of a vaccine candidate, the epitope which binds to the MHC class II HLA DRB1* molecule is essential.

### Analysis of linear and conformational B-cell Epitopes

B-cell epitope is a specific region of an antigen that is detected in a humoral reaction by either a specific B-cell receptor or the antibody. The epitopes of B-cell are classified into two major types: linear or continuous epitopes and conformational or discontinuous B-cell epitopes. Many of the B-cell epitopes have been shown to be of a conformational nature.

### BcPred 1.0 linear B-cell epitopes

The B-cell identification is the crucial step in the design of epitope-based vaccine. Therefore, an In-silico study was conducted via the web server BcPred-1.0 and BepiPred -2.0 to acquire B-cell epitopes in HEV capsid protein.

By using BcPred-1.0 tool, total of 17 epitopes were predicted from the capsid protein of HEV. Among predicted epitopes, 7 epitopes “TAVAPAHDTPPVPDVDSRGA” at position 118–130, “LEFRNLTPGNTNTRVSRYSS” at position 306–325, “WEGTTKAGYPYNYNTTASD” at position 548–567, “AGHRVAISTYTTSLGAGPVS” at position 575–594, “SERLHYRNQGWRSVETSGVA” at position 248–267, “WPQTTTTPTSVDMNSITSTD” at position 212–231 and “PLQDGTNTHIMATEASNYAQ” at position 165–184 were antigenic as predicted by VaxiJen-2.0 server. Due to their elevated antigenicity score, these epitopes may be treated as potent vaccine candidates.

### BepiPred 2.0 linear B-cell epitopes

A total of 15 linear epitopes were observed during the analysis by BepiPred-2.0. Among all these epitopes, after predicting antigenicity by VaxiJen 2.0, the antigenic epitopes were “PTNPFAPDVTAAAGAGPRVRQPARPLGSAWRDQAQRPAAASRRRPTTAGAAPLTAVAPAHDVPDVDSRGAILRRQYNLSTSPLTSSVA” at position 58–148, “PLLPLQDGTNTHIMATEASNYAQY” at position 162–185, “QTTTTPTSVDMNSITS” at position 214–229, “ASEHVIPSERLHYRNQGWRSVETSGVAEEEA” at position 241–271, “RNLTPGNTNTRVSRYSSTARHRLRRGADGT” at position 309–339, “SANGEP” at position 403–408, ”ENAQQDKGIAIPHDIDLG” at position 417–434 “QAVARSLD” at position 508–515,”LSFWEAGTTKAGYPYNYNTTAS” at position 545–566 and “SLGAGPV” at position 587-593.

### Ellipro predicted linear and conformational B-cell epitopes

**Figure 4:**
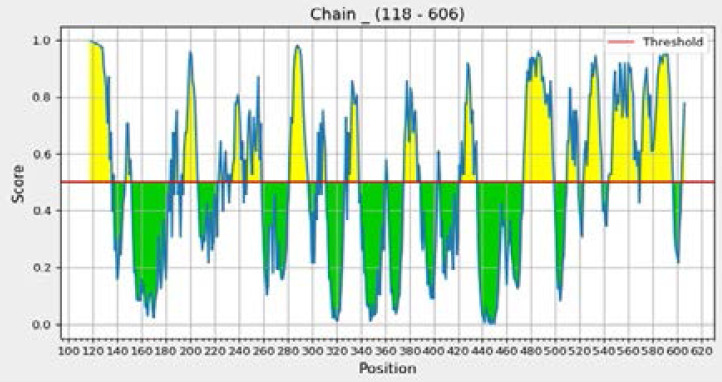
Ellipro 2D score chart.

The threshold was set at 0.5. In the chart (Figure 4) the predictions above the threshold (red line) are positive predictions and negative predictions below the threshold.

### Disco Tope Predicted conformational B-cell Epitopes

In the chart (Figure 5), predictions above the threshold (red line) are positive predictions (green color) and negative predictions below the threshold (shown in pink).

**Figure:**
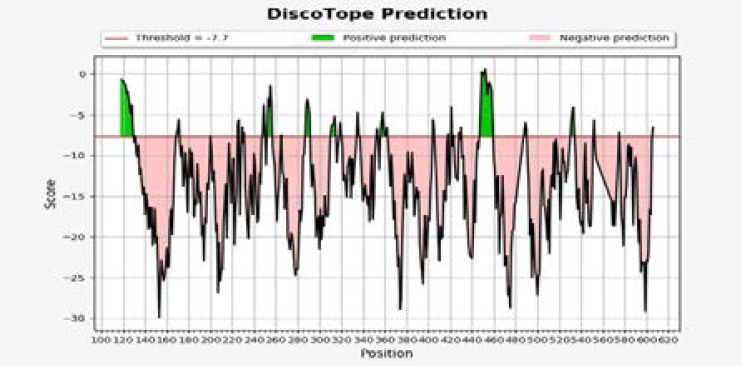
Discotope Chart.

### Three-dimensional analysis of capsid protein

The three-dimensional structure of capsid protein was obtained from RCSB PDB and visualized through Chimera.

## Discussion

This study focuses on one of the important viral infections that infect millions of people worldwide annually. This virus docks onto the intestinal epithelial linings and gets internalized into the gastro-intestinal system, leading towards severe dehydration and clinical manifestations. In the developing countries, there is lack of funding towards exploration of antiviral drugs and vaccine development. Furthermore, there is no mass communication to highlight the disease transmission and preventive measures. Therefore, we aimed towards In-silico prediction of vaccine development against HEV infection while keeping in mind the Global Sustainable Development Goals (SDGs). There are 17 interlinked SDGs, and this study comes under the Goal “Good Health and Well Being”. The endemic situation of HEV not only alarms biologists but highlights the requirement of an effective HEV vaccine. This study suggests highly promising and immunogenic epitopes that elicits B- and T- cells and acts as potential candidates for HEV vaccination. Furthermore, this study reveals ORF2 to be effective for the development of HEV vaccine. ORF2 produces capsid protein that is immunogenic in nature, and which has been used to predict epitopes that could be used as a vaccine.

Previous research focused on ORF2 protein as an important immunogenic part of the HEV that has induced B- cell and T-cell since the genetic analysis of ORF2 showed over 85% similarity between genotypes 1–4 of HEV strains that infect humans 17. In addition, the prediction of HEV capsid protein indicates that it is the target for the neutralization of antibodies 18–20. Therefore, most candidates for HEV vaccines are based on recombinant full-length expressing or trimmed HEV-capsid protein 18. Capsid protein sequence contains 660 amino acids. The 112 to 660 amino acids are gathered into VLPs (virus-like particles) and triggers strong immunological responses. There were also disorganized regions at some specific positions in this protein. Based on antigenicity, CD8+ and CD4+ T-cell epitopes were predicted. Among the 5 CD8+ T-cell epitopes, “WLSLTAAEY” was most antigenic epitope having antigenicity score of 1.5019. In CD4+ T-cell epitopes, the epitopes “WLSTAAEYDQSTYG” “IMATEASNYAQYRVV” “DSQPFAIPYIHPTNP” and “ESRVVIDQYDNQHEQ” peptides showed affinity (IC5O value less than 500nm) with HLA DRB1* allele(s), therefore the strongest binding grooves for the predicted peptides may be HLA DRB1* alleles. It is reported that the most B-cell epitopes (∼90 percent) are not linear or continuous; which means that in the primary structure they are not present 21. In the 3D antigen structure, after protein folding, several residues end up next to each other spatially and are identified by B-cells. Therefore, in the detailed three-dimensional structure of the antigen we predicted the linear and discontinuous B-cell epitopes.

In this study, by BcPred-1.0 7 linear B-cell epitopes were identified. Among these, “LEFRNLTPGNTNTRVSRYSS” was most antigenic with antigenicity score of 1.3600. However, BepiPred-2.0 B-cell epitope data showed that out of 15 linear epitopes, the epitope “SLGAGPV” was most antigenic having antigenicity score of 1.4206. The ORF2 capsid protein of HEV seemed to be an appropriate vaccine target due to its high antigenicity, as suggested by the antigenicity determination through the VaxiJen tool. We picked potent epitopes, using a variety of computer-aided tools.

Conformational or discontinuous B-cell epitopes have been found using the 3D structure of the capsid protein. All the predicted discontinuous epitopes were located on the surface of HEV capsid protein, reflecting the availability of the virus that entered. Discontinuous epitopes were in the form of residues. The residues: ASP, THR and PRO showed the highest scores. The higher score leads to the greater chance of the residue being involved in an epitope. Our research has shown that HEV designed vaccine structure can activate humoral immune responses and lead to the effective activation of pathogen-specific antibodies.

In the history of vaccine development, microbial species (viruses, bacteria etc.) have been used, such as live attenuated vaccines, to induce immune responses that shield the host from infections. Vaccination has therefore proved to be important for the control of multiple lethal infectious diseases. This form of vaccine, however, may contain excessive protein particles for protective immunity activation which may contribute to immunogenic responses. This has contributed to a transition to a new age of vaccine growth through reverse vaccinology. For several viruses, such as Hepatitis C virus (HCV), Human papilloma virus (HPV) and Human immunodeficiency virus (HIV), peptide vaccines have been developed consisting only of epitopes capable of inducing positive, qualifying immune responses mediated by T-cell and B-cell, and other vaccines are also being developed 22–25.

## Figures and Tables

**Figure:**
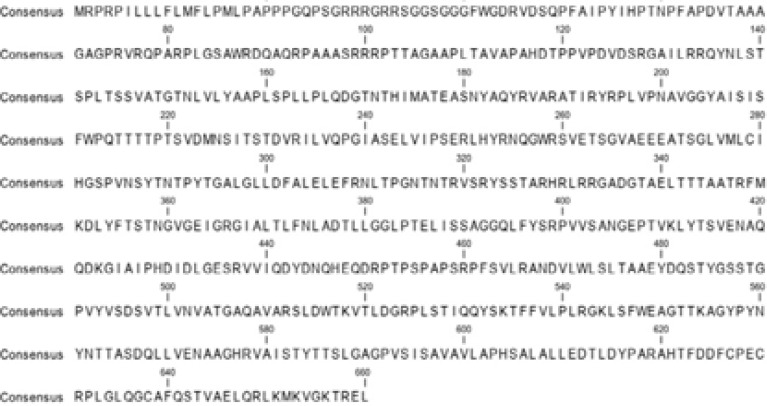
Global consensus sequence.

**Table 2:** CD4+ T-cell Epitopes

ALLELES	EPITOPES	ANTIGENECITY SCORE	IC50(nM)
DRB1*0101	IMATEASNYAQYRVV	0.5240	5.41
	DSQPFAIPYIHPTNP	0.9733	2.15
	WLSLTAAEYDQSTYG	0.8599	0.82
	ESRVVIQDYDNQHEQ	0.6731	40.64
DRB1*0701	IMATEASNYAQYRVV	0.5240	126.77
	WLSLTAAEYDQSTYG	0.8599	47.53

**Table 3:** BcPred linear Epitopes

POSITION	EPITOPES	ANTIGENECITY SCORE
118–130	TAVAPAHDTPPVPDVDSRGA	0.7124
306–325	LEFRNLTPGNTNTRVSRYSS	1.3600
548–567	WEAGTTKAGYPYNYNTTASD	0.6560
575–594	AGHRVAISTYTTSLGAGPVS	0.4702
248–267	SERLHYRNQGWRSVETSGVA	0.8452
212–231	WPQTTTTPTSVDMNSITSTD	0.8709
165–184	PLQDGTNTHIMATEASNYAQ	0.5532

**Table 4:** BepiPred linear Epitopes

POSITION	EPITOPES	ANTIGENECITY
58–148	PTNPFAPDVTAAAGAGPRVRQPARPLGSAWRDQAQRPAAASRRRPTTAGAAPLTAVAP AHDTPPVPDVDSRGAILRRQYNLSTSPLTSSVA	0.5371
162–185	PLLPLQDGTNTHIMATEASNYAQY	0.6789
214–229	QTTTTPTSVDMNSITS	0.7677
241–271	ASEHVIPSERLHYRNQGWRSVETSGVAEEEA	0.7013
309–339	RNLTPGNTNTRVSRYSSTARHRLRRGADGTA	0.5541
403–408	SANGEP	0.6901
417–434	ENAQQDKGIAIPHDIDLG	1.0977
508–515	QAVARSLD	0.5575
545–566	LSFWEAGTTKAGYPYNYNTTAS	0.6282
587–593	SLGAGPV	1.4206

**Table 5:** Ellipro Predicted Linear Epitopes

No.	Chain	Start	End	Peptide	Number of residues	Score
1	A	118	137	DTPPVPDVDSRGAILRRQYN	20	0.86
2	A	474	498	SLTAAEYDQSTYGSSTGPVYVSDSV	25	0.836
3	A	282	297	GSLVNSYTNTPYTGAL	16	0.792
4	A	543	596	GKLSFWEAGTTKAGYPYNYNTTASDQLLVENA AGHRVAISTYTTSLGAGPVSIS	54	0.765
5	A	523	538	GRPLSTTQQYSKTFFV	16	0.747
6	A	193	206	RYRPLVPNAVGGYA	14	0.725
7	A	376	388	ADTLLGGLPTELI	13	0.697
8	A	328	338	RHRLRRGADGT	11	0.693
9	A	421	435	QDKGIAIPHDIDLGE	15	0.679
10	A	509	519	AVARSLDWTKV	11	0.645
11	A	228	258	TSTDVRILVQPGIASEHVIPSERLHYRNQGW	31	0.625
12	A	603	606	PHSA	4	0.601
13	A	305	312	ELEFRNLT	8	0.59
14	A	146	151	SVATGT	6	0.583
15	A	183	189	AQYRVVR	7	0.548

**Table 6:** Ellipro Predicted Discontinuous Epitopes

No.	Residues	Number of residues	Score
1	_:D118, _:T119, _:P120, _:P121, _:V122, _:P123, _:D124, _:V125, _:D126, _:S127, _:R128	11	0.986
2	_:S474, _:L475, _:T476, _:A477, _:A478, _:E479, _:Y480, _:D481, _:Q482, _:S483, _:T484, _:Y485, _:G486, _:S487, _:S488, _:T489, _:G490, _:P491, _:V492, _:Y493, _:V494, _:S495, _:D496, _:S497, _:V498, _:A509, _:V510, _:A511, _:R512, _:S513, _:L514, _:D515, _:W516, _:T517, _:K518, _:V519, _:L521, _:G523, _:R524, _:P525, _:L526, _:S527, _:T528, _:T529, _:Q530, _:Q531, _:Y532, _:S533, _:K534, _:T535, _:F536, _:F537, _:V538, _:P540, _:L541, _:G543, _:K544, _:L545, _:S546, _:F547, _:W548, _:E549, _:A550, _:G551, _:T552, _:T553, _:K554, _:A555, _:G556, _:Y557, _:P558, _:Y559, _:N560, _:Y561, _:N562, _:T563, _:T564, _:A565, _:S566, _:D567, _:Q568, _:L570, _:V571, _:E572, _:N573, _:A574, _:A575, _:G576, _:H577, _:R578, _:V579, _:A580, _:I581, _:S582, _:T583, _:Y584, _:T585, _:T586, _:S587, _:L588, _:G589, _:A590, _:G591, _:P592, _:V593, _:S594, _:I595, _:S596, _:H604, _:S605, _:A606	111	0.757
3	_:T144, _:S145, _:S146, _:V147, _:A148, _:T149, _:G150, _:T151, _:R193, _:P196, _:L197, _:V198, _:P199, _:N200, _:A201, _:V202, _:G203, _:G204, _:Y205, _:A206, _:L235, _:V236, _:Q237, _:P238, _:G239, _:I240, _:A241, _:S242, _:E243, _:G282, _:S283, _:L284, _:V285, _:N286, _:S287, _:Y288, _:T289, _:N290, _:T291, _:P292, _:Y293, _:T294, _:G295, _:A296, _:L297, _:R328, _:H329, _:R330, _:L331, _:R332, _:R333, _:G334, _:A335, _:D336, _:G337, _:T338, _:A376, _:D377, _:T378, _:L379, _:L380, _:G381, _:G382, _:L383, _:P384, _:T385, _:E386, _:I388, _:G392, _:K423, _:G424, _:I425, _:A426, _:I427, _:P428, _:H429, _:D430, _:I431, _:D432, _:L433, _:G434, _:E435	82	0.707
4	_:G129, _:A130, _:I131, _:L132, _:R134, _:Q135, _:A183, _:Q184, _:R186, _:V188, _:R189, _:V245, _:P247, _:S248, _:E249, _:R250, _:L251, _:H252, _:Y253, _:R254, _:N255, _:Q256, _:G257, _:W258, _:E269, _:S273, _:E305, _:E307, _:R309, _:N310	30	0.66
5	_:D223, _:M224, _:N225, _:T228, _:S229, _:T230, _:D231, _:V232, _:R233	9	0.538

**Table 7:** Discotope predicted Discontinuous Epitopes. The positive predictions can be seen in table 3.9. Out of 468 residues, 82 residues showed positive predictions. In the table, each predicted residue's side chain is shown. The table indicates the predicted epitope residues along with the chain id, residue id, residue name, contact number, propensity score and Discotope score

Chain ID	Residue ID	Residue Name	Contact Number	Propensity Score	Discotope Score
A	118	ASP	4	1.339	-0.661
A	119	THR	4	1.339	-0.661
A	120	PRO	5	1.604	-0.896
A	121	PRO	6	1.776	-1.224
A	122	VAL	6	1.651	-1.349
A	123	PRO	7	1.127	-2.373
A	124	ASP	6	0.916	-2.084
A	125	VAL	7	0.589	-2.911
A	126	ASP	8	0.075	-3.925
A	127	SER	10	0.055	-4.945
A	128	ARG	7	-0.36	-3.86
A	129	GLY	11	-1.667	-7.167
A	131	ILE	13	-1.178	-7.678
A	169	GLY	15	0.305	-7.195
A	170	THR	17	1.814	-6.686
A	171	ASN	14	1.466	-5.534
A	200	ASN	12	-1.648	-7.648
A	225	ASN	11	-0.573	-6.073
A	226	SER	11	-0.069	-5.569
A	229	SER	10	-1.52	-6.52
A	231	ASP	11	-1.737	-7.237
A	248	SER	14	-0.1	-7.1
A	249	GLU	12	2.173	-3.827
A	250	ARG	15	0.722	-6.778
A	252	HIS	18	2.145	-6.855
A	253	TYR	13	3.5	-3
A	254	ARG	14	2.931	-4.069
A	255	ASN	10	3.572	-1.428
A	256	GLN	14	3.383	-3.617
A	265	GLY	11	-1.982	-7.482
A	287	SER	15	0.171	-7.329
A	288	TYR	9	0.825	-3.675
A	289	THR	9	1.456	-3.044
A	290	ASN	10	1.103	-3.897
A	291	THR	12	1.198	-4.802
A	310	ASN	15	-0.07	-7.57
A	311	LEU	13	0.102	-6.398
A	312	THR	15	1.222	-6.278
A	313	PRO	18	2.949	-6.051
A	314	GLY	21	5.243	-5.257
A	315	ASN	20	2.83	-7.17
A	319	ARG	23	5.549	-5.951
A	320	VAL	25	5.26	-7.24
A	335	ALA	8	-0.733	-4.733
A	336	ASP	14	-0.402	-7.402
A	353	LEU	18	2.253	-6.747
A	356	THR	12	-0.992	-6.992
A	357	SER	9	-1.303	-5.803
A	358	THR	7	-1.188	-4.688
A	359	ASN	11	-1.092	-6.592
A	360	GLY	13	-1.11	-7.61
A	361	VAL	13	-0.924	-7.424
A	362	GLY	12	-0.649	-6.649
A	404	ALA	10	-0.54	-5.54
A	405	ASN	10	-1.436	-6.436
A	418	ASN	15	0.125	-7.375
A	421	GLN	10	0.977	-4.023
A	423	LYS	16	0.511	-7.489
A	428	PRO	12	-1.231	-7.231
A	430	ASP	14	0.428	-6.572
A	447	HIS	25	5.36	-7.14
A	448	GLU	17	4.959	-3.541
A	449	GLN	13	6.795	0.295
A	450	ASP	17	8.381	-0.119
A	451	ARG	20	9.726	-0.274
A	452	PRO	15	8.167	0.667
A	453	THR	15	6.911	-0.589
A	454	PRO	16	5.357	-2.643
A	455	SER	11	4.135	-1.365
A	456	PRO	9	3.499	-1.001
A	457	ALA	10	3.526	-1.474
A	458	PRO	9	2.496	-2.004
A	459	SER	13	0.438	-6.062
A	489	THR	7	-2.418	-5.918
A	490	GLY	8	-2.787	-6.787
A	531	GLN	10	-0.989	-5.989
A	532	TYR	8	-0.555	-4.555
A	533	SER	7	-0.587	-4.087
A	534	LYS	11	-1.993	-7.493
A	552	THR	7	-2.09	-5.59
A	575	ALA	8	-3.106	-7.106
A	606	ALA	9	-2.023	-6.523

## Conclusion

Effective vaccines are needed globally to control the spread of HEV and improve health status of mankind. To predict highly potential epitopes for vaccine production, an In-silico analysis was carried out towards HEV-ORF2 capsid protein. Based on these immunoinformatics based tools, promiscuous B-cell and T-cell epitopes were predicted for the ORF2 capsid protein that can be selected as possible vaccine candidates against HEV. In addition, these predicted epitopes displayed strong interactions with the MHC-I and MHC-II alleles that shows a tendency towards its therapeutic use in future.
